# The impact of hand hygiene knowledge on self-efficacy among Spanish nursing students: a cross-sectional study

**DOI:** 10.3389/fpubh.2025.1669928

**Published:** 2025-10-09

**Authors:** Ana De Maya-Martínez, Omar Cauli, María del Carmen Giménez-Espert, Cristina Buigues

**Affiliations:** Department of Nursing, University of Valencia, Valencia, Spain

**Keywords:** hand hygiene, infection control, nursing, self-efficacy, education

## Abstract

**Background:**

Hand hygiene (HH) is a good ally to prevent healthcare-associated infections (HAIs). Nevertheless, its incidence continues to concern global bodies such as the World Health Organization (WHO). Nursing student (NS) education will be crucial to reducing HAIs.

**Objective:**

The aim of this current study was to establish the level of HH knowledge among Spanish NS in order to identify gaps in their understanding. We also evaluated self-efficacy among NS as a key strategy for infection control (IC).

**Methods:**

We conducted a cross-sectional study of 483 NS in their second, third, or fourth years in the Faculty of Nursing at University of Valencia (Spain). Participants were recruited by convenience sampling; we collected their sociodemographic data, information on their level of knowledge regarding HH using a WHO questionnaire, and their self-efficacy in IC using a questionnaire based on the Health Belief Model, previously validated and showing moderate to high reliability (ICC = 0.63).

**Results:**

The mean knowledge score was 63.2%, with fourth-year NS achieving significantly higher scores than second-year NS (*p* < 0.001). The poorest results were those for the WHO “5 Moments for Hand Hygiene” item: only 10.6% identified the need for HH before injections, 19.2% recognized alcohol-based hand rub as more effective than handwashing, and just 21.4% responded correctly regarding HH after patient environment contact. Only 18% correctly identified the main source of pathogens causing HAIs. Self-efficacy scores increased significantly with academic experience (*p* < 0.001) and correlated positively with HH knowledge.

**Conclusion:**

In this single-site study, we found that NS had a moderate knowledge of HH, with those in their third or fourth year scoring better than those in their second year. Self-efficacy in IC might play an important role in preventing HAIs and so it is crucial to enhance the effectiveness of HH among NS to improve clinical competence, student self-confidence, and quality of patient care. These data contribute to a body of knowledge that can help improve the NS training curricula endorsed by international organizations with a view to help prevent HAIs.

## 1 Introduction

Healthcare-associated infections (HAIs), which cause substantial morbidity and mortality, remain a significant global public health concern. They result in longer hospital stays and create substantial economic burdens. It is estimated that over 5 million patients are affected by HAIs each year, leading to around 50,000 deaths (1% of all mortalities) per year (1). According to the latest European Centre for Disease Prevention and Control (ECDC) point-prevalence surveys, approximately 7.1% of hospital patients in Europe had at least one HAI, while 3.1% of residents in long-term care facilities were affected (2). The World Health Organization (WHO) reports that between 7 and 15% of hospitalized patients acquire at least one HAI, and 10% of them die, with 20% of these deaths considered preventable (3). Thus, European and global organizations have stressed the need to promote tools and strategies for HAI prevention (1, 3).

Suggested measures include adopting new protocols, improved university-level training, and greater public awareness of hand hygiene (HH) (3). Current actions to reduce HAIs emphasize simple measures such as standard precautions (SPs), particularly HH and the use of personal protective equipment (PPE) (4). Given that the hands of healthcare workers are a frequent source of hospital-acquired infections (1, 5, 6), HH is a critical element of infection control (IC).

Nurses, who are among the largest group of essential healthcare professionals and work in close contact with patients and families, play a central role in IC and prevention (1, 7). It is therefore essential to monitor both the information they receive and their knowledge in these areas. Equally important are nursing students (NS), the future generation of nurses who will assume responsibility for IC and prevention. Hence, their knowledge must be firmly integrated and form the foundation of their training programs (1, 7).

Healthcare-associated IC among NS can be influenced by variables such as academic year and gender (8–13), thereby requiring a personalized educational approach. Published data indicate that while many students have good knowledge and self-efficacy regarding IC, their attitudes toward these practices often remain weak (14). This gap between knowledge and attitude highlights the importance of designing comprehensive curricula for undergraduate NS that not only impart theoretical information, but also encourage the development of positive attitudes toward adherence to IC measures (15). These clinical training courses should integrate interactive teaching methods with continuous and rigorous assessments to ensure that NS not only understand the theory, but also apply their knowledge in real-world situations (14, 16), thus better preparing them for the challenges of the clinical practice.

In addition, it is crucial to consider the role of self-efficacy—understood as confidence in one’s own ability to perform interventions and procedures—in NS training. Self-efficacy is an essential component of NS motivation and the effective application of knowledge in patient care. Hence, self-efficacy not only facilitates the learning process, but also improves the ability of NS to manage complex clinical situations (17–29). The perception of competence and self-efficacy directly influences the quality of care provided and the effectiveness of interventions carried out by future health professionals (20, 21).

The Health Belief Model (HBM) can be used to better understand the impact of self-efficacy in the context of HH training (22). This theoretical framework helps predict and explain health-related behaviors by considering how individual perceptions of risks and benefits affect health decisions and actions. This model focuses on perceptions of susceptibility to disease, severity, expected benefits of adopting healthy behaviors, and perceived barriers to doing so (23). In the context of HH, the HBM helps assess how perceptions of risk and the effectiveness of HH in preventing HAIs influence the attitudes and practices of NS. In addition, self-efficacy is a central component of this model. High self-efficacy can lead to greater adherence to best practices, while low self-efficacy can limit the effective application of such knowledge in real-world clinical situations (17–19).

Nonetheless, the literature analyzing the relationship between HH knowledge and self-efficacy in NS limited. The factors influencing HH practice in NS (15, 24), patient safety in relation to NS self-efficacy (21), and the self-efficacy of health professionals in implementing HH (25) have been previously studied. However, studies with large sample sizes that specifically explore the relationship between HH knowledge and self-efficacy in NS at different points in their undergraduate education are still scarce (26). Thus, we aimed to establish the level of HH knowledge among Spanish NS in order to identify areas for improvement in nursing curricula endorsed by international organizations which set standards and recommendations for health education (1, 4).

As part of the internal evaluation of our nursing curricula, we compared HH knowledge among NS at different stages of their nursing degree. We hypothesized that NS self-efficacy would vary depending on their level of HH knowledge and aimed to identify possible knowledge gaps where further training in HH could be useful (1, 4). To gain a deeper understanding of how perceptions of self-efficacy and other beliefs affect the implementation of HH measures, we used the HBM as the theoretical framework. The main objective of this study was to evaluate the relationship between HH knowledge and the academic year of undergraduate NS at the University of Valencia (Spain) in order to identify weaknesses in the curricula that require reinforcement. The secondary objective was to evaluate the association between NS HH knowledge and self-efficacy.

## 2 Methods

### 2.1 Study design

A cross-sectional study was carried out to evaluate the level of HH knowledge and self-efficacy among NS in the Faculty of Nursing and Podiatry at the University of Valencia (Spain). The participants were all students enrolled in the second, third, and fourth academic years of the bachelor’s nursing degree who consented to take part after receiving information about the study. Confidentiality and anonymity of the data were guaranteed. The questionnaires, which included sociodemographic data such as age, nationality, gender, and marital status, were sent via a Google Form link to all the students via a virtual classroom message. Similar numbers of students in each academic year were invited to participate in the study: 258 in the second year (response rate *n* = 188, 72.86%), 247 in the third year (response rate *n* = 144, 58.29%), and 225 in the fourth year (response rate *n* = 139, 61.77%).

### 2.2 Evaluation of hand hygiene knowledge

The WHO hand hygiene knowledge questionnaire for health-care workers (1) was used to evaluate NS knowledge of HH. We employed the Spanish version of this questionnaire, authorized by the Spanish Ministry of Health (30) and previously used with Spanish NS (26, 31), consisting of 10 items. Item 1 covers training in the 3 years prior and item 2 covers compliance with the routine use of an alcohol-based hand rub (ABHR), but these items are excluded from the scoring. Items 3–10 assessed the NS level of knowledge of HH and included 25 questions covering pathogen transmission routes, sources of infection, indications for HH for patients and healthcare workers, the effectiveness of ABHRs, minimum rubbing time required, and clinical situations and risk factors for pathogen colonization. Each correct answer was scored as 1 and each incorrect answer as 0, yielding a total score ranging from 0 to 25, with higher values indicating better knowledge.

### 2.3 Evaluation of self-efficacy in infection control practices

Self-efficacy in IC practices was measured using a survey based on the Health Belief Model (HBM), a theory-based framework used to predict health-related behaviors and assess the perceptions and knowledge of IC practices among clinical professionals in hospitals. The original instrument comprises 6 subscales that mirror the 6 HBM constructs: (1) susceptibility, (2) severity, (3) benefits, (4) barriers, (5) self-efficacy, and (6) cues to action. Each item is scored on a 5-point Likert scale from 1 (strongly disagree) to 5 (strongly agree), where higher scores reflect stronger self-efficacy in IC.

In our study, we used only the 6 items related to self-efficacy in IC to address our primary study aim. The items were as follows: (1) I engage in good infection control practices; (2) I seek information on infection control practices; (3) Engaging in proper infection control measures is important to me; (4) I follow infection control recommendations regularly; (5) I often use hand sanitiser while working in the health care setting; (6) Hand sanitisers are as effective as hand washing in controlling infections. The total IC self-efficacy score ranges between 6 and 30 points, with higher scores indicating higher self-efficacy.

The validity of the original instrument was previously evaluated by an external review panel (including a licensed nurse, a doctoral-level nurse with IC experience, a medical epidemiologist, and 2 doctoral-level health science professors). The HBM has been shown to be internally reliable and has a Cronbach alpha ranging from 0.65 to 0.81 (32). The 6 items we used were translated according to the procedure recommended by Beaton et al. (33), obtaining a Cronbach alpha of 0.630 (standardized *α* = 0.684) and an intraclass correlation coefficient (ICC) of 0.630. According to the proposed ICC repeatability thresholds (≥ 0.75 = excellent, 0.4–0.74 = fair to high, and ≤ 0.39 = poor) (34), the scale demonstrated moderate to high repeatability.

### 2.4 Sample size determination

To calculate the required sample size, we hypothesized, *a priori*, a moderate association (*r* = 0.4) between HH knowledge and self-efficacy. A two-tailed test, *α* = 0.05, 95% confidence interval (95% CI), *β* = 0.20, and power of (1 − *β*) = 0.80 were also assumed. Anticipating a 10% dropout rate due to incomplete questionnaires, the required sample size was 52 students. This estimate was based on the classification of coefficients as weak (< 0.3), moderate (0.3–0.7), or strong (> 0.7) (35, 36), and application of the ARCSINUS approximation (37). A posteriori, with the observed correlation coefficient of 0.15, the required sample size was 386; the final sample size achieved was 483 NS.

### 2.5 Data collection

To simplify data collection and facilitate participation, we designed a self-administered questionnaire using Google Forms. The questionnaire contained three sections: sociodemographic data, HH knowledge (1), and IC-related self-efficacy. We sent an informative e-mail to the second-, third-, and fourth-year NS and posted an explanatory video in their virtual classroom outlining the project and inviting them to complete the anonymous questionnaire. The participants were informed that their involvement in the study would not affect their academic performance. To minimize bias, none of the researchers involved in the study took part in the recruitment of participants, except to provide information about the study. The NS participated between September 2023 and December 2023.

### 2.6 Statistical analysis

Quantitative variables were expressed as the mean ± standard deviation (SD). Comparisons were made with Student *t*-tests for parametric data or Mann–Whitney U tests for non-parametric data. Qualitative data were compared using chi-squared tests or Fisher exact tests. Correlations among quantitative variables were examined using Pearson correlation coefficients and Spearman rank correlations. Multiple linear regression analyses were conducted to examine the associations between the total HH knowledge score and the independent variables of interest (age, gender, course year, and IC self-efficacy), while accounting for potential confounding participant characteristics. Results were reported as regression coefficients (*β*) with their 95% CIs and *p*-values. Statistical analyses were conducted using SPSS software (version 28, IBM Corp., Armonk, NY), with *p* < 0.05 considered statistically significant.

## 3 Results

### 3.1 Characteristics of the study sample

A total of 483 individuals participated in this study, 188 (38.9%) second year, 146 (30.2%) third year, and 149 (30.8%) fourth year NS. The mean participant age was 22.2 ± 0.26 years (standard error of the mean) and their ages ranged between 18 and 58 years. Regarding their gender, most participants, 406 in total (84.1%), identified as female and 73 (15.1%) identified as male, while a small percentage identified as genderfluid (1; 0.2%), pangender (2; 0.4%), or other (1; 0.2%). In terms of their living arrangements, most (253; 52.4%) lived with their parents, 165 (34.2%) lived in shared housing, 24 (5%) lived with their partners, 18 (3.7%) lived with their partners and children, 3 (0.6%) lived with other family members or close friends, and 7 (1.4%) resided only with their children.

### 3.2 Hand hygiene knowledge

**Figure 1:**
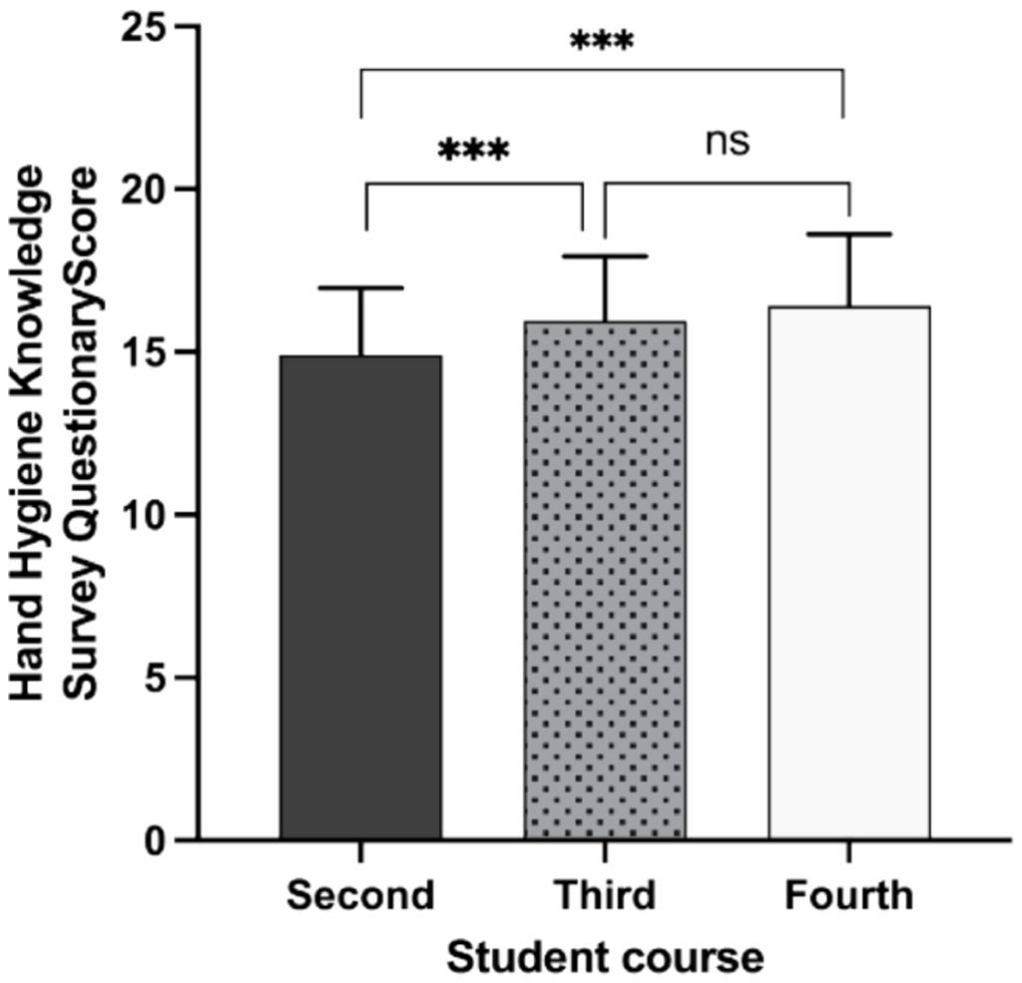
Hand hygiene knowledge among nursing students from different undergraduate nursing undergraduate course study years. Comparison of the scores obtained in the hand hygiene knowledge questionnaire from students in the second, third, or fourth-year course of the 4-year bachelor’s degree in nursing at University of Valencia (Spain). Statistically significant differences are indicated by asterisks. ns, non-significant difference. *** (*p <* 0.001).

Table 1 summarizes the correct and incorrect responses given by NS regarding HH knowledge, based on the WHO questionnaire (1). Differences were observed across academic years (*p <* 0.001, Figure 1), with fourth-year NS scoring significantly higher than second-year NS. The overall mean correct score was 63.2%, though performance varied widely by item.

**Table 1:** The WHO hand hygiene knowledge questionnaire for health-care workers (1).

Questions	Frequency (%) of answer		
	Second year (*N* = 188)	Third year (*N* = 146)	Fourth year (*N* = 149)
1. Did you receive formal training in hand hygiene in the last 3 years?			
Yes	119 (63.3)	141 (96.6)	146 (98.0)
No	69 (36.7)	4 (3.4)	3 (2)
2. Do you routinely use alcohol-based hand rub for hand hygiene?			
Yes	61 (32.8)	70 (47.9)	108 (72.5)
No	127 (67.6)	76 (52.1)	41 (27.5)
3. Which of the following is the main route of cross-transmission of potentially harmful germs between patients?			
**a) *Health-care workers’ hands when not clean***	96 (51.1)	89 (61.0)	120 (80.5)
b) Air circulating in the hospital	21 (11.2)	18 (12.3)	10 (6.7)
c) Patients’ exposure to colonized surfaces (i.e., beds, chairs, tables, floors)	57 (30.3)	26 (17.8)	12 (8.1)
d) Sharing non-invasive objects (i.e., stethoscopes, pressure cuffs, etc.) between patients	14 (7.4)	13 (8.9)	7 (4.7)
4. What is the most frequent source of germs responsible for healthcare-associated infections?			
a) Hospital’s water system	1 (0.5)	0 (0)	2 (1.3)
b) Hospital air	33 (17.6)	35 (24.0)	30 (20.1)
**c) *Germs already present on or within the patient***	33 (17.6)	31 (21.2)	25 (16.8)
d) Hospital environment (surfaces)	121 (64.4)	80 (54.8)	92 (61.7)
5. Which of the following hand hygiene actions prevents transmission of germs to patients?			
a) Before touching a patient			
Correct answer is ‘**Yes**’	185 (98.4)	144 (98.6)	147 (98.7)
b) Immediately after a risk of a body fluid exposure			
Correct answer is ‘**No**’	25 (13.3)	32 (21.9)	34 (22.8)
c) After exposure to the immediate surroundings of a patient			
Correct answer is ‘**No**’	37 (19.7)	29 (19.9)	37 (24.8)
d) Immediately before a clean/aseptic procedure			
Correct answer is ‘**Yes**’	173 (92.0)	143 (97.9)	145 (97.3)
6. Which of the following hand hygiene actions prevents transmission of germs to healthcare workers?			
a) After touching a patient			
Correct answer is ‘**Yes**’	173 (92.0)	137 (93.8)	144 (96.6)
b) Immediately after a risk of body fluid exposure			
Correct answer is ‘**Yes**’	171 (91.0)	142 (97.3)	142 (95.3)
c) Immediately before a clean/aseptic procedure			
Correct answer is ‘**No**’	152 (80.9)	115 (78.8)	107 (71.8)
d) After exposure to the immediate surroundings of a patient			
Correct answer is ‘**Yes**’	174 (92.6)	140 (95.9)	143 (96.0)
7. Which of the following statements on alcohol-based hand rub and hand washing with soap and water are true?			
a) Hand rubbing is more rapid for hand cleansing that hand washing			
Correct answer is ‘**True**’	134 (71.3)	113 (77.4)	124 (83.2)
b) Hand rubbing causes skin dryness more than hand washing			
Correct answer is ‘**False**’	166 (88.3)	127 (87.0)	122 (81.9)
c) Hand rubbing is more effective against germs than hand washing			
Correct answer is ‘**True**’	45 (23.9)	24 (16.4)	26 (17.4)
d) Hand washing and hand rubbing are to be performed in sequence			
Correct answer is ‘**False**’	153 (81.4)	90 (61.6)	74 (49.7)
8. What is the minimum time needed for alcohol-based hand rub to kill most germs on your hands?			
***20 s***	94 (50.0)	94 (64.4)	101 (67.8)
3 s	5 (2.7)	1 (0.7)	1 (0.7)
1 min	64 (34.0)	32 (21.9)	37 (24.8)
10 s	25 (13.3)	19 (13.0)	10 (6.7)
9. Which type hand hygiene method is required in the following situations?			
a) Before palpation of the abdomen			
**Rubbing**	84 (44.7)	89 (61.0)	94 (63.1)
Washing	94 (50.0)	51 (34.9)	51 (34.2)
None	10 (5.3)	6 (4.1)	4 (2.7)
b) Before administering an injection			
**Rubbing**	48 (25.5)	29 (19.9)	55 (36.9)
Washing	138 (73.4)	116 (79.5)	94 (63.1)
None	2 (1.1)	1 (0.7)	0
c) After emptying a bedpan			
**Rubbing**	33 (17.6)	11 (7.5)	10 (6.7)
Washing	149 (79.3)	134 (91.8)	136 (91.3)
None	6 (3.2)	1 (0.2)	3 (2)
d) After removing examination gloves			
**Rubbing**	46 (24.5)	68 (46.6)	78 (52.3)
Washing	137 (72.9)	77 (52.7)	59 (46.3)
None	5 (2.7)	1 (0.7)	2 (1.3)
e) After making a patient’s bed			
**Rubbing**	61 (32.4)	61 (41.8)	76 (51)
Washing	121 (64.4)	83 (56.8)	71 (47.7)
None	6 (3.2)	2 (1.4)	2 (1.3)
f) After visible exposure to blood			
Rubbing	69 (36.7)	29 (19.9)	22 (14.8)
***Washing***	118 (62.8)	117 (80.1)	127 (85.2)
None	1 (0.5)	0	0
10. Which of the following should be avoided, as associated with an increased likelihood of colonization of hands with harmful germs?			
a) Wearing jewelry			
Correct answer is ‘**Yes**’	167 (88.8)	141 (96.6)	144 (96.6)
b) Damaged skin			
Correct answer is ‘**Yes**’	180 (95.7)	140 (95.9)	139 (93.3)
c) Artificial fingernails			
Correct answer is ‘**Yes**’	178 (94.7)	142 (97.3)	144 (96.6)
d) Regular use of hand cream			
Correct answer is ‘**No**’	115 (61.2)	63 (43.2)	90 (60.4)

Correct answers are indicated in bold.

The weakest results concerned the WHO ‘5 Moments for Hand Hygiene’ guidelines. For Moment 1 (before touching a patient), only 10.6% recognized that HH is required before administering an injection (item 9b). For Moment 2 (before an aseptic procedure), just 19.2% knew that ABHR is more effective than hand washing with soap and water (item 7c). For Moment 3 (after body fluid exposure risk), 19.3% answered correctly regarding HH immediately after exposure (item 5b), and 27.4% after emptying a bedpan (item 9c). For Moment 4 (after touching a patient), 41.1% identified the need for HH after glove removal (item 9d). For Moment 5 (after touching patient surroundings), only 21.4% responded correctly about HH after contact with the patient’s environment (item 5c). Knowledge was also very limited regarding the main source of germs causing HAIs (item 4), with only 18% answering correctly.

Second-year NS obtained the lowest HH knowledge scores, followed by third-year students. Pearson chi-squared tests revealed significant differences by study year for three items: (1) whether they had received formal training in HH in the 3 years prior (*p* < 0.001); (2) whether they regularly used an ABHR for HH (*p* < 0.001); and (3) whether they knew the main route of cross-transmission of potentially pathogenic microorganisms in healthcare settings (*p* < 0.001).

No significant differences were found for questions (4), (5), or (6) by study year. Question (4) addressed the most frequent source of germs causing HAIs (*p* = 0.568). Question (5) referred to the HH actions that prevent the transmission of microorganisms to patients (a), before touching a patient (*p* = 0.977); (b), immediately after the risk of exposure to body fluids (*p* < 0.065); (c), after contact with the immediate environment of the patient (*p* = 0.454); and (d), immediately before a clean/aseptic procedure (*p* = 0.139). Question (6) inquired about the HH actions that prevent the transmission of microorganisms to healthcare professionals. No differences by year were found for items (a), after touching a patient (*p* = 0.206); (c), immediately before a clean/aseptic procedure (*p* = 0.129); or (d), after contact with the immediate environment of the patient (*p* = 0.276). However, significant differences were found for item (b), immediately after the risk of exposure to body fluids (*p* = 0.040).

For question (7), which asked which statements about using ABHR preparations and washing hands with soap and water were true, significant differences were found in two of the four items by study year: (a), hand rubbing is faster than hand washing (*p* = 0.035) and (d), whether sequential hand washing and rubbing is recommended (*p* < 0.001). No significant differences were observed for items (b), regarding whether rubbing causes more dry skin than hand washing (*p* = 0.220) or (c), whether rubbing is more effective against germs than hand washing (*p* = 0.166). Significant differences were also observed by NS study year in the responses to question (8), which asked about the minimum rubbing time required with ABHR to eliminate germs from hands (*p* < 0.001).

Significant differences were again observed by NS study year for question (9), which asked about the type of HH required (rubbing, washing, or none) in different situations: (a), before abdominal palpation (*p* = 0.001); (b), before administering an injection (*p* = 0.004); (c) after emptying a bedpan (*p <* 0.002); (d) after removing gloves (*p* < 0.001); (e), after making a patient bed (*p* = 0.003); and (f), after visible exposure to blood (*p* < 0.001). For question (10), which addressed practices associated with increased risk of hand colonization by pathogenic microorganisms, significant differences by NS study year were noted for (a) wearing jewelry (*p* = 0.03) and (d) regular use of hand cream (*p* < 0.001). No significant differences were detected for (b) skin lesions (*p* = 0.03) or (c) the use of artificial fingernails (*p* < 0.001).

### 3.3 Self-efficacy in infection control practices

The mean self-efficacy score was 21.7 (SD = 3.25) in the second year (*n* = 188), 23.1 (SD = 3.06) in the third year (*n* = 146), and 23.2 (SD = 2.52) in the fourth year (*n* = 149; *p* < 0.001). These findings suggest an upward trend in perceived self-efficacy regarding IC as students progressed through their academic training.

### 3.4 Relationship between HH knowledge and self efficacy in infection control

**Figure 2:**
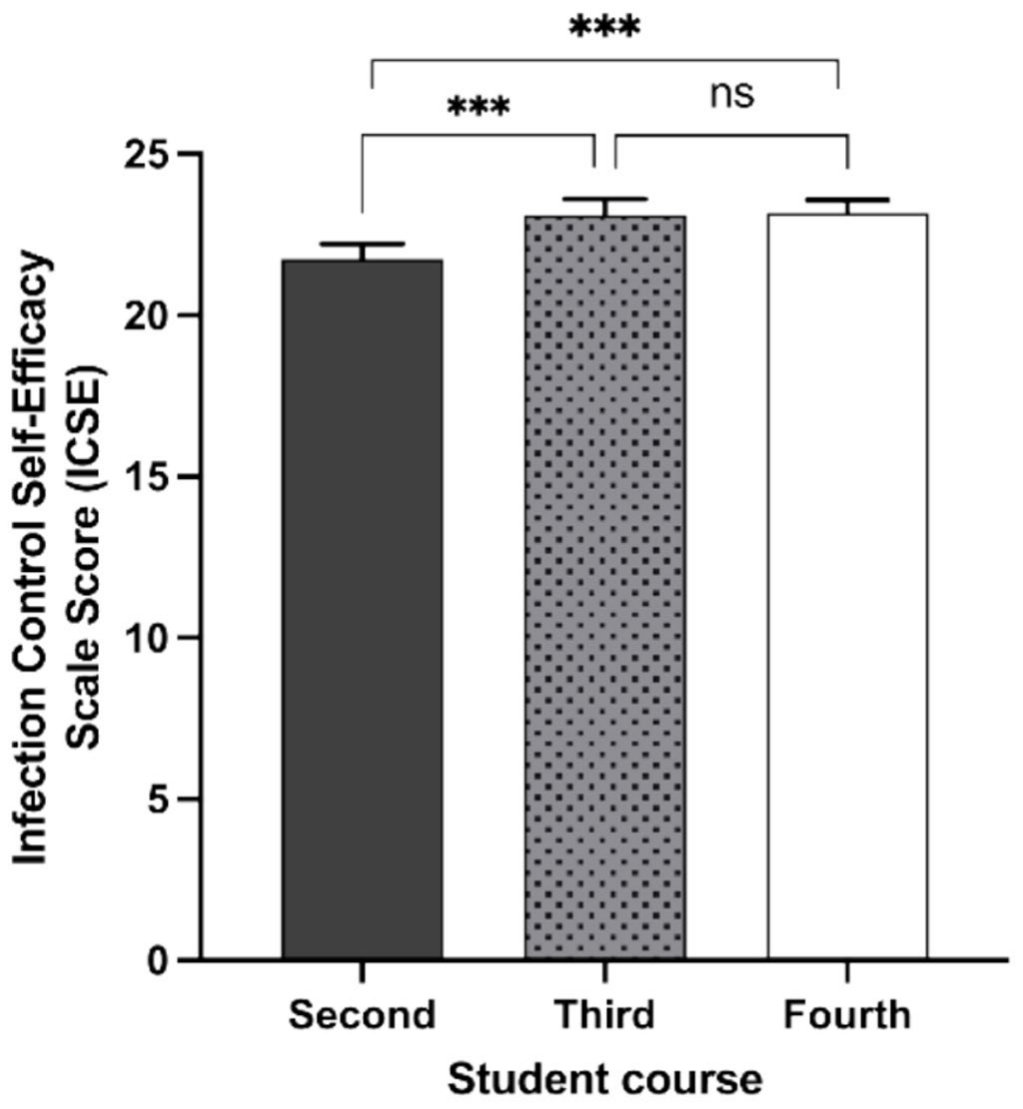
Infection control self-efficacy in nursing students from different undergraduate nursing course years. Comparison of the scores obtained in the infection control self-efficacy questionnaire from students in the second, third, or fourth year course of the 4-year bachelor’s degree in nursing at University of Valencia (Spain). Statistically significant differences are indicated by asterisks. ns, non-significant difference. *** (*p <* 0.001).

**Figure 3:**
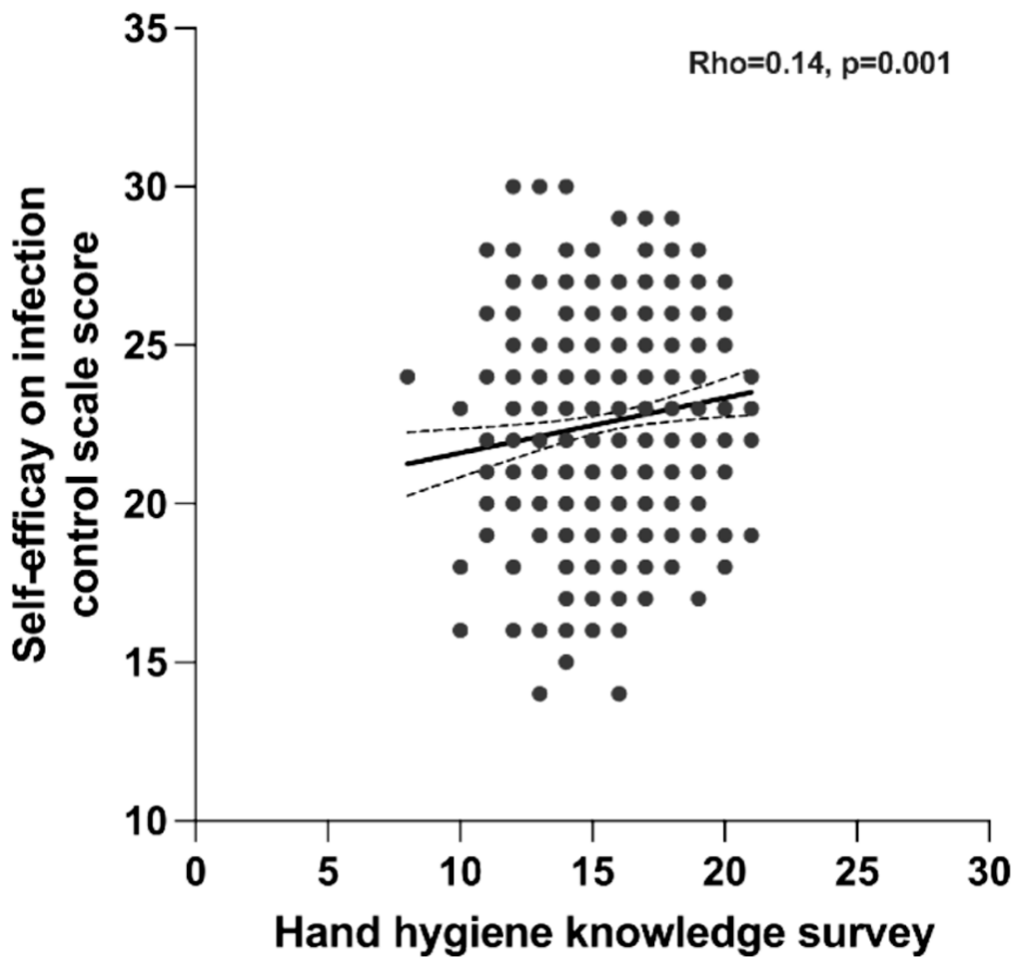
Correlation between self-efficacy related to infection control and HH knowledge. The association between scores obtained in the hand hygiene knowledge questionnaire and the infection control self-efficacy questionnaire from all the students participating in the study. The rho correlation coefficient and *p*-values are indicated.

When analyzing the differences in IC self-efficacy among NS by study year (Figure 2), fourth-year students scored significantly higher (*p* < 0.001) than those in the second or third years. In addition, as shown in Figure 3, there were significant correlations between the HH knowledge survey results and IC self-efficacy score (Rho = 0.148, *p* < 0.001, Spearman correlations).

### 3.5 Linear analysis

A linear regression was conducted to examine associations between HH knowledge survey scores and potential predictors (age, gender, course year, and IC self-efficacy). The study year was significantly associated with HH knowledge (*β* = 0.74, *p* < 0.001, 95% CI [0.51, 0.96]). No significant associations were found with age (*β* = −0.03, *p* = 0.069, 95% CI [−0.06, 0.002]), gender (*β* = −0.33, *p* = 0.087, 95% CI [−0.70, 0.05]), or IC self-efficacy score (*β* = 0.06, *p* = 0.055, 95% CI [−0.001, 0.13]).

A second regression examined HH knowledge in relation to the six individual IC self-efficacy items (Table 2). Significant associations were observed for two items: the belief that it is important for NS to adopt appropriate IC measures (*β* = 0.39, *p* = 0.037, 95% CI [0.02, 0.76]) and frequent use of hand sanitizer in healthcare settings (*β* = 0.38, *p* = 0.003, 95% CI [0.13, 0.63]). No significant associations were found for the remaining items.

**Table 2:** Association between hand hygiene knowledge and self-efficacy for infection control items.

Variable	Standardized beta coefficients	*t*	*p*-value	95% confidence interval (lower limit, upper limit)
I engage in good infection control practices	0.19	0.36	0.71	−0.26, 0.38
I seek information on infection control practices	−0.91	−1.87	0.06	−0.42, 0.01
Engaging in proper infection control measures is important to me	**0.17**	**2.08**	**0.04**	**0.23, 0.78**
I follow infection control recommendations regularly	−0.01	−0.16	0.87	−0.63, 0.30
I often use hand sanitiser while working in healthcare settings	**0.14**	**2.96**	**0.03**	**0.13, 0.63**
Hand sanitisers are as effective as hand washing in controlling infections	0.37	7.98	0.425	−0.94, 0.22

Significant *p* values are indicated in bold.

## 4 Discussion

The aim of this study was to establish the level of HH knowledge among Spanish NS and identify areas of deficiency that warrant greater attention in nursing curricula endorsed by international organizations. In addition, perceived self-efficacy in hand washing as a key IC strategy was evaluated, providing information that can guide training to reinforce both theoretical knowledge and confidence in applying appropriate practices.

Spanish NS acquire skills and knowledge over their four-year undergraduate program. In the first 2 years, learning is largely theoretical, complemented by simulations, practical classroom activities, and basic clinical practice. In the third and fourth years, NS apply their knowledge in real-world settings, carrying out more advanced care practices. This progression ensures that, by the end of their training, these future professionals are capable of implementing correct hygiene and hand washing behaviors.

In this work, the overall mean score across all study years was moderate (63.2%), comparable to those reported in Greek NS (60.4%) (38) and Saudi NS (68.7%) (39). Our findings highlight variation in NS HH knowledge across course years and survey items, and also in its relationship with IC-related self-efficacy. NS with more experience (third and fourth years) reported higher rates of HH training in the preceding 3 years (96.6 and 98.0%, respectively) compared to only 63.3% of second-year NS. Similar patterns have been observed elsewhere: Blomgren et al. reported increasing HH knowledge across semesters among Swedish NS (40), and a study of second- to fourth-year NS Turkey found that Hand Hygiene Practices Inventory scores improved across course years (27). In Norwegian nursing homes during the COVID-19 pandemic, lower education levels were associated with decreased HH adherence among healthcare workers, suggesting a link between staff training duration and HH compliance (41).

In our study, NS knowledge regarding question 4 of the WHO questionnaire—“the most frequent source of germs responsible for healthcare-associated infections”—was insufficient, with few students in any academic year answering correctly and an overall mean correct response rate of only 18%. This clearly indicates that NS were not fully aware of key HAI prevention and IC measures. Similarly, Rafaqat and Ahmed (42) also found inadequate HAI and IC knowledge, noting that participants lacked sufficient information. Another study recommended improving compliance with healthcare guidelines by increasing NS awareness of the high incidence and costs of HAIs (43). In summary, knowledge in this critical area was low and requires reinforcement.

Despite high knowledge rates regarding HH before patient contact or after an aseptic procedure—with correct response rates exceeding 92% across all three course years—gaps were evident in situations involving contact with potentially risky fluids or patient environments. In these cases, correct response rates were very low, ranging from 13.3 to 24.8% depending on the course year. Adherence to HH after contact with patient environments was particularly insufficient, suggesting that NS often neglect HH in these situations. This is concerning given the evidence that hospital environments play a key role in HAI transmission (44–46, 65). Previous studies have also shown that HH following contact with patient surroundings is among the most commonly missed HH moments for healthcare professionals, with compliance rates of only 36% (44, 45). These findings underscore the need to reinforce both awareness and training in this critical aspect of infection control (46, 65).

Our data also highlight misconceptions about the use and effectiveness of ABHR, with many students believing that handwashing is more effective for eliminating germs (the overall mean correct response rate for this item was only 19%). Nevertheless, we observed that ABHR use increased as NS advanced through their training, suggesting, as noted by Kingston et al. (47), that knowledge gaps may be a key barrier to adoption. Misconceptions should therefore be corrected by emphasizing WHO guidance that ABHR should be applied for 20–30 s when hands are not visibly soiled, and that routine use does not cause skin damage. Incorporating a dedicated module on correct ABHR technique and indications into HH curricula could reinforce both NS self-efficacy and compliance.

Beyond misconceptions about ABHR, many participants did not correctly differentiate between the indications for hand rubbing versus hand washing, reflecting broader gaps in knowledge about the appropriate application of each technique. Importantly, these results represent knowledge scores rather than observed compliance so they cannot be directly equated with behavioral adherence. In our sample, higher knowledge scores were obtained for HH after contact with blood (80.1 and 85.2% correct among third- and fourth-year students, compared with 62.8% among second-year students).

Similar patterns of self-reported compliance have been described in previous studies, such as Sundal et al. (48), Kingston et al. (46), and Ceylan et al. (27). In contrast, our students showed the greatest uncertainty regarding HH after administering an injection, removing gloves, or making a patient bed, consistent with findings from Nair et al. (49) and Thakker & Jadhav (50). These specific gaps—particularly those related to WHO Moment 5 (“after contact with patient surroundings”)—suggest that interventions should go beyond general HH training to include targeted, scenario-based education and practical reinforcement in the situations most frequently neglected.

Regarding the role of self-efficacy, we found a significant association with HH knowledge, suggesting that the more prepared and educated NS are, the better their ability to apply HH correctly and manage complex clinical situations. However, the correlation coefficient (*r* = 0.148) between these factors indicated only a small effect, which should be interpreted with caution until potential moderating variables are identified that could strengthen this relationship. Consistent with our findings, Lewis & Thompson (51) reported that NS perceived proper HH as positively influencing IC, benefiting patients, hospitals, and the students themselves. Similarly, a study of Korean NS found a positive correlation between HH knowledge and HH performance (52).

This is crucial, as perceiving these benefits encourages students to adopt appropriate IC practices. Self-efficacy also influences academic success (53–55), enhancing the ability of NS to overcome challenges in clinical practice, achieve goals, and deliver high-quality patient care (56). Conversely, self-efficacy is linked to burnout and exhaustion, reducing the likelihood of academic success (57). Evidence suggests that self-efficacy in NS can be strengthened through mastery experiences, vicarious learning, social persuasion, and awareness of psychological states—approaches that build confidence in problem solving and clinical competence (54).

It is essential to address the development of NS self-efficacy during training, particularly given reports of rising burnout in the nursing profession (58). Strengthening NS self-efficacy can enhance safety and confidence in clinical settings. In our study, self-efficacy appeared to reflect the confidence and willingness of NS to perform IC practices, with significant associations observed for two of the six scale items: recognizing the importance of adopting adequate IC measures and frequently using ABHR in healthcare settings. This contrasts with findings from Jeong et al. (59). To foster good healthcare practice, a solid theoretical foundation in HH and HAI prevention must be provided in early in nursing curricula, followed by skills development and reinforcement through practical training (60). In this sense, various researchers have proposed different methodological strategies to consolidate IC-related knowledge and promote the development of self-efficacy in this domain (38, 46, 61–64).

The main limitations of this study are that it was conducted at a single university and employed a cross-sectional design, which does not allow causal inferences to be made. In addition, the use of convenience sampling limits the generalisability of the findings because the participants may not fully represent the broader student population and so the results should be interpreted with caution. A participation rate of 72% was obtained from second-year students, 58.29% from third-year students, and 61.77% from fourth-year students, meaning that more than half of all nursing courses were represented. Non-responder bias was acceptable for second- and fourth-year NS, but moderate for third-year NS, which limits the generalisability of comparisons across study years.

This study relied exclusively on self-reported data, which may be affected by social desirability bias. This makes it difficult to determine the accuracy of responses without observational or objective measures of HH. Thus, while our findings identify gaps in HH knowledge and perceived self-efficacy in IC, they cannot be taken as direct evidence of actual adherence. Therefore, future research should incorporate observational methods to assess whether these factors translate into consistent HH behavior.

Another limitation lies in the translation and adaptation of the self-efficacy scale for IC practices in our context. In addition, the relatively low internal consistency observed for the self-efficacy subscale (Cronbach *α* = 0.63) warrants caution. This may be partly explained by the reduced number of items in the subscale and the specific characteristics of the study population. Nevertheless, the intraclass correlation coefficient (ICC = 0.63) indicated moderate-to-high repeatability, suggesting that the construct retains a reasonable level of stability. Future studies should therefore employ random sampling to improve generalisability, involve multiple universities, and adopt longitudinal designs that allow causal relationships to be established.

## 5 Conclusion

This study showed that NS at the University of Valencia (Spain) had a moderate level of HH knowledge. Students in the latter part of the nursing undergraduate degree (third and fourth years) scored better than those at the beginning of their training. This indicates that the knowledge of HH acquired throughout the degree is generally solid, although there are aspects that should be improved, such as knowledge about the appropriate use of ABHR and the importance reinforcing HH practice after contact with patient environments to prevent HAIs.

In addition, there was a significant link between self-efficacy and IC, highlighting the importance of enhancing self-efficacy among NS, thereby benefitting their clinical competence, self-confidence, and capacity to deliver high-quality of patient care. Considering that NS are future healthcare professionals, it is vital that they acquire adequate knowledge during undergraduate training. Thus, we encourage implementing new university-level strategies to improve learning regarding HH, IC, and HAI prevention, thereby better preparing students for professional practice.

## Data Availability

The original contributions presented in the study are included in the article/supplementary material, further inquiries can be directed to the corresponding author.
